# Efficient Deep Learning Based Hybrid Model to Detect Obstructive Sleep Apnea

**DOI:** 10.3390/s23104692

**Published:** 2023-05-12

**Authors:** Prashant Hemrajani, Vijaypal Singh Dhaka, Geeta Rani, Praveen Shukla, Durga Prasad Bavirisetti

**Affiliations:** 1 Computer and Communication Engineering, Manipal University Jaipur, Jaipur 303007, Rajasthan, India; 2 Department of Computer Science, Norwegian University of Science and Technology, 7034 Trondheim, Norway

**Keywords:** MobileNet V1, Long Short Term Memory, obstructive sleep apnea, single-lead ECG, Gated Recurrent Unit

## Abstract

An increasing number of patients and a lack of awareness about obstructive sleep apnea is a point of concern for the healthcare industry. Polysomnography is recommended by health experts to detect obstructive sleep apnea. The patient is paired up with devices that track patterns and activities during their sleep. Polysomnography, being a complex and expensive process, cannot be adopted by the majority of patients. Therefore, an alternative is required. The researchers devised various machine learning algorithms using single lead signals such as electrocardiogram, oxygen saturation, etc., for the detection of obstructive sleep apnea. These methods have low accuracy, less reliability, and high computation time. Thus, the authors introduced two different paradigms for the detection of obstructive sleep apnea. The first is MobileNet V1, and the other is the convergence of MobileNet V1 with two separate recurrent neural networks, Long-Short Term Memory and Gated Recurrent Unit. They evaluate the efficacy of their proposed method using authentic medical cases from the PhysioNet Apnea-Electrocardiogram database. The model MobileNet V1 achieves an accuracy of 89.5%, a convergence of MobileNet V1 with LSTM achieves an accuracy of 90%, and a convergence of MobileNet V1 with GRU achieves an accuracy of 90.29%. The obtained results prove the supremacy of the proposed approach in comparison to the state-of-the-art methods. To showcase the implementation of devised methods in a real-life scenario, the authors design a wearable device that monitors ECG signals and classifies them into apnea and normal. The device employs a security mechanism to transmit the ECG signals securely over the cloud with the consent of patients.

## 1. Introduction

In humans, sleeping is a universal functionality, comprising thirty-three percent of their life. The outcome of sleep deficiency results in dysfunction in most body functions. A sleep disorder may present as a problem associated with aberrant sleep deficiency [1,2]. Sleep apnea is a severe problem brought on by sleep disturbances. According to The American Academy of Sleep Medicine (AASM), it is a sleep disorder in which patients experience breathing pauses while they are asleep [3]. The three different types of sleep apnea are central sleep apnea (CSA), obstructive sleep apnea (OSA), and complex sleep apnea (CSA) [4]. Airflow during sleep is said to stop for 10 s due to OSA, which is caused by a blockage in the upper airway [5,6]. The brain’s respiratory center regulates breathing and creates airflow disruptions while sleeping, which causes central sleep apnea. The phrase “complex sleep apnea” refers to a condition in which a patient suffers from obstructive and central sleep apnea [2]. An obstruction in the upper respiratory tract causes OSA, a common problem for humans. Drowsiness upon awakening, daytime tiredness, and snoring indicates OSA [7,8]. According to [9], a study was conducted on Indians in 2015, where nearly 93% of people found insomnolence. The prevalence of OSA has been observed higher in the country’s western region. According to the report, OSA varies between 4.4% and 19.7% for males and 2.5% to 7.4% for females [10]. OSA is also becoming more well-recognized as a primary cause of high blood pressure, cerebrovascular disease, and cardiovascular disease [6,11]. The Apnea-Hypopnea Index (AHI) is a clinical assessment of OSA severity that counts apnea and hypopnea occurrences during a specific time while sleeping [7]. Polysomnography (PSG) is the “gold standard” for identifying OSA in clinical terms [8,11]. To detect sleep problems, patients must wear several wires and sensors on their bodies for one or more nights and rely on specialized laboratories and people. PSG is an expensive and time-consuming procedure that is tough to implement. As a result, researchers have proposed several single-lead signals-based OSA detection methods because single signals such as the ECG can pinpoint the occurrence of OSA in diagnosed patients due to a decrease in blood oxygen levels, forcing the cardiovascular system to work harder to maintain an adequate oxygen level throughout the body, forcing the cardiovascular system to work harder to maintain proper oxygen level throughout the body. In an earlier study, the majority of researchers relied on standard machine learning algorithms and CNN models, both of which lacked the ability to extract features under varying sizes and channels [8,9,12] To improve the accuracy of the classification of obstructive sleep apnea, the proposed research seeks to optimize the features extracted from the convolutional neural network derived from the electrocardiogram (ECG) signal and associate it with a recurrent neural network (LSTM). This is the novel aspect of the research. Our key contributions are as follows:

1. Two OSA detection models are proposed, first using MobileNet V1, which offers improved accuracy over the state-of-the-art on the same data set.
2. The second OSA detection model is proposed by integrating MobileNet V1 with two different RNNs (LSTM and GRU) used separately to provide two distinct deep-learning models. This model offers the highest accuracy, specificity, and sensitivity over the state-of-the-art on the same data set.
3. A secured wearable device to detect and classify ECG signals of the patient being apneic or not. A security mechanism assures the data received from the sensors is left open to any mishandling.

The rest of this paper is laid out as follows. The related works will be described in Section 2. The preprocessing methods, training parameters, and proposed algorithms are all described in Section 3. In Section 4, the dataset and results are shown. Section 5 contains the conclusion.

## 2. Related Works

Most single-lead OSA (Obstructive Sleep Apnea) detection research points to the use of pulse oximetry and ECG-based signals. The extraction of information (e.g., frequency and time domain, and other variables), and finding patterns and trends are used to determine and predict OSA occurrence accurately. A cumulative study of previous work conducted on the Physionet Apnea-ECG database [13], using Lead-II ECG signals (35 withheld + 35 released) is shown in Table 1.

Changyue Song et al. has used the Discriminative Hidden Markov Model (HMM) to detect OSA from ECG signals [14]. However, this was unable to point to the severity level of the OSA episode. The result is limited to a Boolean value with failure to elaborate thereof. Another study based on DNN and HMM was performed by Kunyang Pan et al. using a single-lead ECG signal [7]. Different classifiers increased the method’s performance [Support vector machines (SVM), ANN, HMM]. The decision fusion algorithm and Newton method were also used [11]. In contrast, the disadvantage of this research is the absence of classification and illness detection.

For extricating multiple features from the RR intervals (RRIS) sequence, Qi Shen et al. [8] used a methodology based on the multiscale dilation attention 1-D convolutional neural network model, a multiscale feature extraction algorithm, and classifiers with weighted loss and time-dependence (WLTD) [15]. Single-lead ECG signals were employed, which were helpful since they are more useful in wearable devices than in medical monitoring systems [8]. Unfortunately, the network model struggled to automatically extract characteristics from the original ECG data, necessitating substantial manual intervention. Deep Learning is employed for classification while Long Short-Term Memory (LSTM) recurrent networks are applied for feature extraction. The 2D-CNN model and the LSTM were used to recover the spatial and temporal properties [16].

A single-lead ECG model, proposed by Kaicheng et al., will be used for unsupervised feature learning to detect sleep apnea. The frequential stacked sparse auto-encoder (FS-SAE) and the time-dependent cost-sensitive (TDCS) classification served as the model’s foundation, and the Hidden Markov Model (HMM) was used to create it [17].

Gregoire Surrel et al. proposed a wearable device that was an energy-efficient system through time-domain analysis using single-channel ECG signals. This device can transfer its results to an internet website for constant monitoring and tracking of the progression of the ailment due to its Bluetooth connection [18].

In addition, Singh et al. [19] employed a continuous wavelet transform (CWT) to construct a two-dimensional scalogram image from each minute of the ECG segment. It analyzed CNN and AlexNet models.

Tao Wang et al. devised a method that employs a time window artificial neural network to model the temporal dependency between ECG signal segments without requiring any previous preconceptions about training data distribution [12].

Some authors have also worked on OSA detection using multiple other techniques. Table 2 shows some of these results. We can see that a maximum accuracy of 97.5% was achieved by [7] with the help of a random forest classifier. Whereas the lowest of 80.5% was exhibited by [20] who used Single channel ECG and hybrid ML Models. The above studies have used the Physionet Apnea dataset with various combinations of released and withheld data apart from 35 + 35 to produce results.

**Table 1:** Cumulative study of previous work conducted on the Physionet Apnea-ECG database, using Lead-II ECG signals (35 withheld + 35 released).

Year	Authors	Technique Used	Application	Features	Accuracy
2017 [21]	Gutta, S., Cheng, Q., Nguyen, H.D. and Benjamin, B.A.	Hidden Markov Model	OSA Detection	Temporal dependence on signals using Hidden Markov Model (HMM)	82.33%
2018 [9]	Wang, L., Lin, Y. and Wang, J.	DNN and HMM using single lead ECG signal	OSA Detection	Hidden Markov Model (HMM) and Deep Neural Network (DNN)	85%
2018 [18]	Surrel, G., Aminifar, A., Rincon, F. and Murali, S.	SVM	OSA Detection	RR Interval and RS amplitude time series	88.2%
2019 [19]	Singh, S.A. and Majumder, S.	AlexNet model, CNN	OSA Detection	Time-Frequency Scalogram	86.22%
2019 [22]	Lu, C. and Shen, G.	Time window artificial neural network (TW-MLP)	SA Detection	RR Interval and RPeak Amplitude	87.3%
2020 [17]	Feng, K., Qin, H., Wu, S., Pan, W. and Liu, G.	Unsupervised feature learning, single lead ECG	SA Detection	Frequential stacked sparse autoencoder (FSSAE)	85.1%
2021 [8]	Shen, Q., Qin, H., Wei, K. and Liu, G.	Multiscale deep neural network	OSA Detection	Multiscale Dilation Attention Convolution	89.4%

**Table 2:** A comparative study of multiple other methods on the Physionet Apnea-ECG database, using Lead-II ECG signals other than (35 withheld + 35 released).

Year	Authors	Technique Used	Application	Features	Accuracy
2020 [23]	Bozkurt, F., Uçar, M.K., Bozkurt, M.R. and Bilgin, C.	Single channel ECG and hybrid ML Model	Detection of abnormal respiratory events with obstructive sleep apnea	Fisher Feature Selection Algorithm, Principal Component Analysis	85.12%
2020 [5]	Mc-Clure, K., Erdreich, B., Bates, J.H.T., McGinnis, R.S., Masquelin, A. and Wshah, S.	Multiscale Deep Neural Network and 1DCNN with wireless sensors	OSA Detection	1-D Convolutional Neural Network	86%
2019 [20]	Stretch, R. et al.	Machine Learning (KNN, SLR, ANN, SVM, Gradient boosted decision tree, etc.)	OSA Detection	Least Absolute Shrinkage and Selection Operator (LASSO) and Ridge Regression	80.5%
2019 [19]	Singh, S.A. and Majumder, S.	AlexNet	Sleep Apnea Detection	Deep Neural Network	86.22%
2019 [24]	Liang, X., Qiao, X. and Li, Y.	CNN and LSTM	OSA Detection	RR Interval	99.8%
2017 [21]	Gutta, S., Cheng, Q., Nguyen, H.D. and Benjamin, B.A.	Vector valued Gaussian processes (GPs)	OSA Detection	RR Interval	82.33%
2015 [7]	Song, C., Liu, K., Zhang, X., Chen, L. and Xian, X.	Hidden Markov Model	OSA Detection	RR Interval	97.1%

## 3. Methods and Material

### 3.1. Dataset Description

The proposed paper uses the apnea-ECG database from PhysioNet, which contains 70 single-lead ECG recordings, to train and evaluate the suggested techniques. The data were gathered at a polling rate of 100 Hz, with a resolution of 16 bits and nominally 200 A/D units per millivolt, and a resolution of 16 bits with the least significant byte first in each pair. This results in a database having 34,428 min of data and 34,230 min of annotations. The length of each recording varies from just less than 7 h to approximately 10 h. The occurrence of OSA in each individual record is predetermined using other metrics and signals (e.g., apnea annotations manually derived based on synchronously recorded respiration and related signals, computer-generated QRS annotations, chest, and polysomnographic (PSG) data), but no determination on the occurrence of hypopnea or apnea was made. There are two types of datasets among the 70 records obtained: the withheld dataset (nomenclature a01–a20, b01–b05, c01–c10) and the released dataset (nomenclature x01–x35). As a result, the withheld set was used to train the model, while the released set was utilized to validate it [13].

### 3.2. Pre-Processing of Data

**Figure 1:**
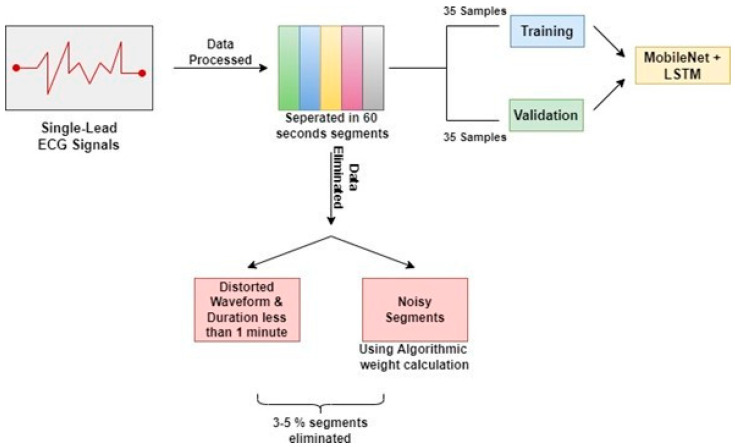
Preprocessing of Physionet Apnea-ECG database.

Single-lead ECG measurements were performed on patients for 7 to 10 h. Following that, the ECG data were separated into 60-s segments for analysis. Signals containing distorted waveforms and samples with a total duration of less than one minute were eliminated from the data set utilized to construct the model. Furthermore, noisy segments in the remaining dataset were discarded using an algorithmic weight calculation method. The auto-correlation functions of the parts were measured with a 60 s time delay using the aforementioned procedure for elimination. Only about 3 to 5 percent (1404) of the segments were eliminated during the overall cleaning procedure. The dataset includes 70 patients and is split into two parts: the first portion contains the data of 35 patients for the purpose of training, and the second part includes the data of 35 patients for the purpose of testing and validation to generate the study’s results. Figure 1 illustrates the steps involved in the preprocessing of the data.

### 3.3. Training Parameters

#### 3.3.1. Optimizer

An optimizer is a function/algorithm to modify the attributes of a neural network (e.g., weights of epochs, learning rate) and serves to minimize the loss function, thus improving accuracy. The weight is initialized using a variety of techniques and modified with each epoch according to the update equation [25,26].

Adaptive first- and second-order moment estimation is used in the stochastic gradient descent method known as Adam optimization. Instead of employing the standard stochastic gradient descent technique, it may regularly modify weights in the network based on training statistics [26]. The gradient descent approach can be sped up by using the gradients’ exponentially weighted average. The gradient descent approach can be sped up by using the gradients’ exponentially weighted average. Stochastic gradient descent operates a single learning rate for all weight changes (alpha) [27,28]. Throughout the training, the pace, as mentioned earlier, remains constant. As learning progresses, the learning rate of each network weight (parameter) is adjusted individually. Adam extends the capabilities of stochastic gradient descent by combining the benefits of two previous optimization methodologies: the Root Mean Square Propagation, which maintains per-parameter learning rates that are tailored based on a set of boundaries (e.g., how quickly it is changing), and the Adaptive Gradient Algorithm/Momentum, which improves performance on problems with sparse gradients (such as natural language processing and computer vision problems) [29]. Both non-stationary and stationary problems can be tackled using this method.

#### 3.3.2. Activation Function

An activation function in a neural network explains how the weighted sum of a neuron’s input is converted into output by calculating the sum and adding its own bias to it in a layer of the neural network [30,31]. This is important for converting what would essentially be a linear regression model into a capable neural network with nonlinear inputs that can learn and perform more complex tasks, and it works in conjunction with the model’s use of the backpropagation weight initialization technique because the gradients are supplied along with the error to update the weights and biases, and is thus important for converting what would essentially be a linear regression model into a capable neural network with nonlinear inputs that can learn and perform more complex tasks [30,32]. As a result, it is critical to the network’s success, and multiple activation functions are frequently employed for different portions of the model. The rectified linear activation function (ReLU), a piecewise linear function used in this work, returns zeros for negative input and returns the input directly for positive input. It can be expressed in Equation (1)

*f*(*x*) = *max*(0, *x*) (1)

where, *f*(*x*) equals *x* when *x* is less than zero, and *f*(*x*) equals *x* when *x* is higher than or equal to zero. As a result, it is the default activation for building multilayer perceptron and convolutional neural networks since it resolves the vanishing gradient problem, enabling models to train more quickly and perform better. Stochastic gradient descent with backpropagation of errors is needed to train deep neural networks. Although it appears to behave and look like a linear function, it actually possesses nonlinear properties that can be utilized to uncover intricate data correlations [29]. Additionally, it reduces saturation while increasing sensitivity to overall activity. When employing backpropagation to train a neural network, the function is linear for values greater than zero, which has many of the same advantages as a linear activation function. Negative values are always output as zero, indicating that the function is nonlinear.

#### 3.3.3. Weight Initializer

Prior to training neural network models on a dataset, weight initialization is used to specify the initial values for the parameters which are repeatedly updated with the training of the model [33]. This process of weight initialization has a significant effect on the classification of a model. Its goal is to avoid layer activation outputs from becoming worthless due to explosion or disappearance during deep neural network forward passes. To update weights, the proposed model employs the backpropagation mechanism, whereby current weights are dependent on prior weights. This prohibits setting the weights to zero, which would cause gradients to burst. The alternative outcomes are represented by a normal distribution of weights with a mean of 0 and a standard deviation of 1.

#### 3.3.4. Loss Function

In the context of model refinement, a loss function is a function that maps variable events onto an arbitrary real number representing a specific cost associated with the event, i.e., the loss function is responsible for computing the graphed distance between the expected output and the actual output of an algorithm to evaluate the algorithm’s performance. Stochastic gradient descent is used to train neural networks, which necessitates the usage of a loss function. It is in charge of selecting the most appropriate weights and condensing a model’s attributes into quantifiable performance metrics that, when increased, indicate an increase in the model’s accuracy along with improved robustness [34]. Categorical cross-entropy is a loss function used to assess the difference between two probability distributions in multiclass classification issues. It is just a softmax activation with some cross-entropy loss tossed in for good measure. Cross-entropy loss is mathematically described in Equation (2),

(2) $$CE=\sum_{j}^{c}t_{j}\log\;\left(s_{j}\right)$$

where *t_j_* and *s_j_* are the ground truth and the CNN score for each class *j* in *c*. Table 3 displays the hyperparameters that were taken into consideration while tuning the hybrid deep-learning models.

### 3.4. Training Procedure

#### 3.4.1. Architecture of MobileNet V1

MobileNet is a reduced depth-wise structure incorporating a convolutional layer that may be used to differentiate details based on two controllable features that efficiently transition between the parameter’s Accuracy and latency [35]. The core structure consists of multiple abstraction layers, each of which is a component of distinct convolutions that seem to be the quantized configuration that analyses the complexity of a typical problem in great detail [36]. This architecture is ideal for use on mobile devices and computers with limited computational power because it is highly efficient with a small number of features (for example, palmprint recognition) and requires a lot less computational work than conventional CNN models. Detailed images depicting the aforementioned architecture can be referred to in the article available online [37]. It also reduces network size [38] and along with depiction [39].

#### 3.4.2. Recurrent Neural Network

**Figure 2:**
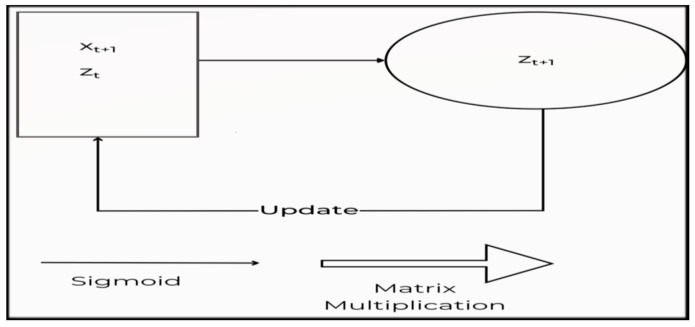
The construction of a conventional RNN.

A recurrent neural network (RNN) is a machine learning algorithm that can evaluate the linear input of samples once at a time. RNN adapts to volatile data in sequential information of different sizes [40,41]. Figure 2 depicts the building of a traditional RNN. The present data X*_t_* are delivered to the node z(*t* + 1), along with the hidden layer’s hidden state data from the earlier stage ht shown in Figure 2. As a consequence, RNN is a looped neural network that changes over time to allow information to persist.

Given an input sequence X = [x1, x2, …, xT], an RNN specifies a series of hidden states *h_t_* given by Equation (3)

*h_t_*_+1_ = *ψ*(*Z_t_*_+1_) = *ψ*(*W_h_h_t_* + *W_x_ X_t_* + *b*) (3)

**Figure 3:**
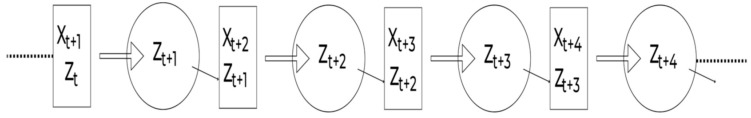
A complex RNN structure.

An RNN may be conceived of as numerous replicas of the same network, each forwarding a message to a successor, as represented in Figure 3.

RNNs were created to model sequential data. However, because of the common difficulty of vanishing/exploding gradients, training RNNs using stochastic gradient descent (SGD) is fairly difficult [42]. The bursting gradient issue is very simple to solve when the gradients’ norm is restricted. On the other hand, architectural advancements such as LSTM, GRU, and iRNN/uRNN [21,42] may aid with the issue of vanishing gradients.

#### 3.4.3. Long Short-Term Memory

Several theoretical and practical articles on this kind of Recurrent Neural Network (RNN) have been published since the first LSTM research in 1997 [43]. Several researchers remarked upon these exceptional outcomes involving sequential datasets such as text, language modeling, video, and speech-to-text transcription [21,44]. Influenced mainly by its excellent standards discussed in the literature, numerous readers in academic and commercial contexts seek to understand more about the Long Short-Term Memory network (therefore, “the LSTM network”) to assess its relevance to their research or practical application. Several RNN and LSTM network frameworks are efficiently and production readily implemented in all leading accessible machine-learning platforms. The ease of use and low cost of development and testing help many experimenters, particularly those new to RNN/LSTM systems.

On the other hand, others are keener in delving further into every component of the system’s working. Taking the longer route allows you to develop intuition that will assist you with data preparation, troubleshooting, and adapting an open-source module to fulfill the requirements of your academic project or commercial solution. In most cases, one such task expands to include reading a slew of documents, blog entries, and implementation guides to gain an “A to Z” knowledge of the system’s core principles and operations, only to discover that the vast majority of the resources leave one or more critical questions about the fundamentals unanswered. Unrolling is frequently offered as lacking reasoning. The Recurrent Neural Network (RNN), a generic category of neural network that precedes and includes the LSTM network as an example, is commonly presented without reason. Furthermore, the training equations are frequently removed, making the observer bewildered and needing additional materials to reconcile the numerous notations used.

The LSTM network tackles the problem of unstable gradients by enabling the network to learn long-term dependencies. Zaremba’s [45] LSTM will be demonstrated in-depth. Assume “U” is the linear convolution transformation of the current input, and denotes the linear convolution transformation of the prior output. The input, cell, and hidden states are represented by the steps “n”, “Xn”, “cn”, and “hn”. The hidden state hn and cell state cn are computed for the current input xn as shown in Equations (4)–(6),

*p* = tan *h* ((*U_xn_* + *Wh*_*n*−1_) + *b*) (4)

*C_n_* = *f* #*C*_*n*−1_ + *i*#*j* (5)

*h_n_* = *o*# tan *c_n_* (6)

where b represents all bias terms and # signifies elementwise multiplication. The forget gate, input gate, and out the gate are represented by the letters *f*, *i*, and *o* as shown in Equation (7),

*f*, *i*, *o* = *σ*((*U_xn_* + *Wh_n−_*_1_) + *b* (7)

**Figure 4:**
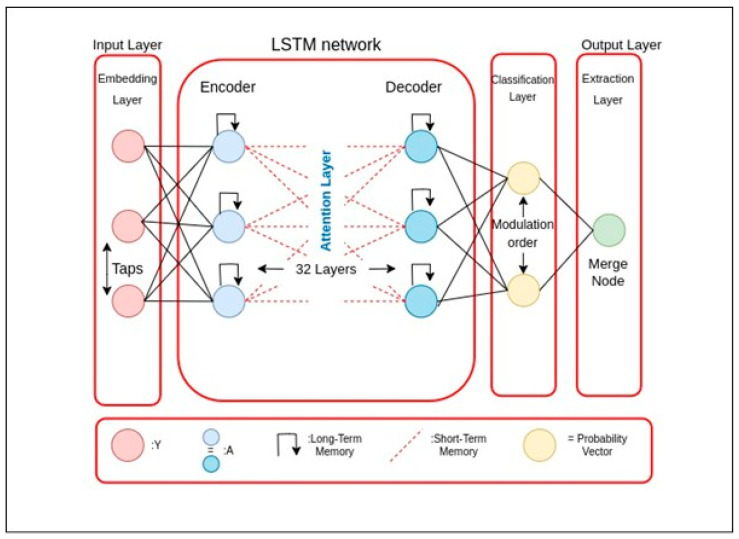
Basic LSTM Structure.

A number between 1 and 0 is returned by the sigmoid activation function. Finally, “hn” represents the LSTM layer’s output at iteration n. The fundamental construction of the LSTM memory block is demonstrated in Figure 4.

X Liang et al. [21] used the unfolding of Bidirectional LSTM networks (BLSTM). Two distinct LSTM networks govern forward. The proposed and backward motion in BLSTM. This structure analyzes combining prior and upcoming sequential information in real-time. Figure 4 depicts the LSTM fundamental in its most basic form. W Yang et al. [46] used the MIT-BIH polysomnography database [13] to test their technique. They took 14 recordings, including one that had a signal detected using a nasal thermistor. However, there are no annotations for start and end positions in this database. It only offers epoch annotations for 30 s. The events were tagged as 30 s epoch annotations, and the database’s single respiration signal was sent to the LSTM network. The method’s recall on the MIT-BIH polysomnography database is 90.0%, 87.1%, and 83.2%, respectively, for normal, apnea, and hypopnea episodes.

#### 3.4.4. Gated Recurring Unit

After MobileNet V1, the Gated Recurrent Unit (GRU), an update of the standard, was used in place of the LSTM. There is no defined cell state in GRUs. There is just a hidden state. GRUs can be trained more quickly because of their more straightforward architecture. GRUs are able to store and filter the data with the help of their update and reset gates. Keeping the crucial information and transmitting it to the network’s subsequent time steps rather than discarding the fresh input each time solves the vanishing gradient problem [47].

#### 3.4.5. Proposed Algorithm

##### MobileNet V1

Depthwise separable convolutions are used in order to implement MobileNet V1. Following are the two parts of a depthwise separable convolution namely, Depthwise convolution which is a distinct map for each input channel of a single convolution. As a result, the total output channels are equal to the total input channels and pointwise convolution with a 1 × 1 kernel size that merely combines the depthwise convolution’s features as shown in Algorithm 1.
**Algorithm 1**: Training Procedure of the MobileNet V1begin (1) Select “n” data as training samples in order. (2) Apply the Depthwise Separable Convolution operation with the form of D*_K_* × D*_K_*. (3) After Step 2, apply Pointwise Convolution to reduce the dimension. (4) Apply Batch Normalization and ReLU after each convolution. (5) Introduce Width Multiplier *α*. To control the total number of channels or channel depth, M converts to *α* M. The value of *α* is ranging from 0 to 1, with standard settings of 1, 0.75, 0.5, and 0.25. (6) for *α* = 1, it is the baseline MobileNet V1. The number of parameters and the computing cost can both be lowered quadratically by roughly *α*2, with Accuracy dropping off smoothly from *α* = 1 to 0.5, until *α* = 0.25 which is too small. To control the network’s input values Resolution Multiplier “*ρ*” was applied, which ranges from 0 to 1. (7) for *ρ* = 1, it is the baseline MobileNet V1. end

##### MobileNet V1 + LSTM

The proposed model intends to combine MobileNet V1 with LSTM and produce outputs as a combination of the two, given ECG signal is the input fed to the model. The working of MobileNet V1 with LSTM can be seen in Algorithm 2.
**Algorithm 2**: Training Procedure of the MobileNet V1 + LSTMbegin (1) Select 35 data as training samples in order. (2) Df^2^ × M × Dk^2^ (3) M × N × Df^2^ (4) Apply Batch Normalization and ReLU after each convolution (5) Introduce Width Multiplier (6) for *α* = 1, it is the baseline MobileNet V1 (7) for *ρ* = 1, it is the baseline MobileNet V1. LSTM (8) Input h*_t_*_1_, C*_t_*_1_, and x*_t_*. (9) Input to first sigmodal layer h*_t_*_1_, x*_t_*. (10) Multiply output of forget gate [0, 1] × C*_t_*_1_. (11) Input to second sigmodal layer h*_t_*_1_, x*_t_*. (12) The tanh layer creates a vector C*_t_*. (13) Pointwise multiplication i*_t_* × C*_t_*. (14) Forget gate output multiplied with previous cell state f*_t_* × C*_t_*_1_. (15) The output is determined by the sigmoid layer, while the tanh layer modifies it in the range of [1]. (16) To obtain the cell’s output h*_t_*, the resultant of both layers is multiplied with point-wise multiplication. end

##### MobileNet V1 + GRU

**Figure 5:**
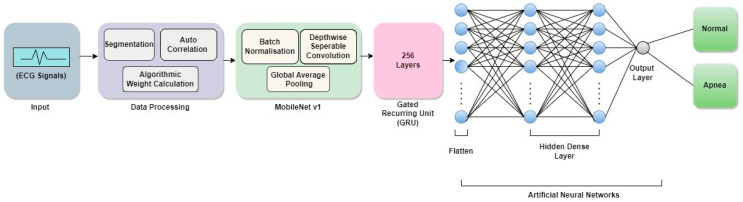
Schematic outline of the proposed architecture of MobileNet V1 + GRU.

The proposed model intends to combine MobileNet V1 with GRU and produce outputs as a combination of the two, the given ECG signal is the input fed to the model. The model architecture and Algorithm are quite similar to the above-mentioned approach the only difference is the use of GRU instead of LSTM. Figure 5 shows the schematic architecture of the proposed MobileNet V1 + GRU model. The working of MobileNet V1 with LSTM can be seen in Algorithm 3.
**Algorithm 3**: Training Procedure of the MobileNet V1 + GRUbegin (1) Select 35 data as training samples in order. (2) Df^2^ × M × Dk^2^ (3) M × N × Df^2^ (4) Apply Batch Normalization and ReLU after each convolution (5) Introduce Width Multiplier. (6) for *α* = 1, it is the baseline MobileNet V1. (7) for *ρ* = 1, it is the baseline MobileNet V1. GRU (8) Input h*_t_*_1_, C*_t_*_1_, and x*_t_*. (9) Input to first sigmodal layer h*_t_*_1_, x*_t_*. (10) Calculate update gate z*_t._* (11) Calculate reset gate r*_t_*, for model to decide on past information to forget, using which new memory content will store relevant information from past. (12) Network will then calculate h*_t_* vector holding information for current unit and pass it down to network. An update gate is needed to determine what to collect from current memory content h’*_t_* and what from previous step h*_t−_*_1_. (13) Pointwise multiplication i*_t_* × C*_t_*. (14) To obtain the cell’s output h*_t_*, the resultant of both layers is multiplied with point-wise multiplication. end

## 4. Experimental Results

### 4.1. Experimental Setup

The code implementation was carried out using the TensorFlow framework on desktop PCs with an Intel(R) Core(TM) i7-6500U CPU running at 2.50 GHz, an Nvidia 940 M GPU with a computing capacity of 5.0, and 16 GB of RAM. The training was conducted over a limited number of epochs. The entire training was conducted on a workstation with an Nvidia RTX 2080 GPU with a computation capability of 8.60 and 11 GB of GPU RAM. To obtain the highest training and testing accuracy, each Model was trained for 100 epochs.

### 4.2. Evaluation Index

To formulate the various values needed for functioning of the model, researchers employed the evaluation methods such as accuracy, specificity, sensitivity, precision and recall as described below in Equations (8)–(12) [48]: The evaluation index analysis on MobileNet V1 is shown in Table 4. Removing four layers from the original MobileNet V1 model also helps to reduce the model’s computation time. Furthermore, the evaluation index analysis on MobileNet V1 +LSTM, and MobileNet V1 + GRU is shown in Table 5 and Table 6.

(8) $$Accuracy=\frac{TP+TN}{TP+TN+FP+FN}\;$$

(9) $$Specificity=\frac{TN}{TN+FP}\;$$

(10) $$Sensitivity=\frac{TP}{TN+FP}\;$$

(11) $$Precision=\frac{TP}{TP+FP}\;$$

(12) $$F1=\frac{2\times Precision\times Recall}{Precision+Recall}\;$$

where,

TP = True Positive
TN = True Negative
FP = False Positive
FN = False Negative

### 4.3. Results

The data of 70 patients used in this study have been evaluated using MobileNet V1 + LSTM and MobileNet V1 + GRU. Table 7 shows a comparative analysis for each segment of OSA Detection with various models, where Model 1 represents the implementation of MobileNet V1, Model 2 represents the implementation of MobileNet V1 and LSTM, and Model 3 represents the implementation of MobileNet V1 and GRU.

A model is a true positive when it accurately predicts the positive class. A true negative, on the other hand, is a result for which the model correctly predicts the negative class. When the model wrongly predicts the positive class, a false positive occurs. A false negative is a result where the model incorrectly predicted the negative class. Table 8 shows the result of the enhanced MobileNet V1 + LSTM model (Model 2) and MobileNet V1 + GRU model (Model 3) for per segment classification.

**Figure 6:**
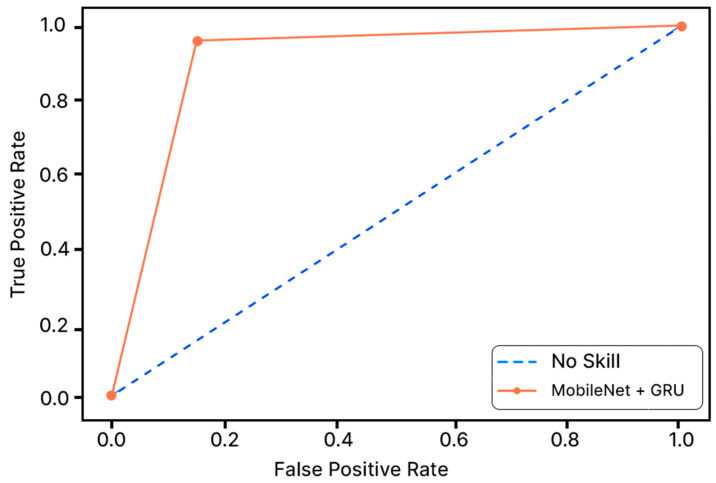
AUC Curve of MobileNet + GRU.

**Figure 7:**
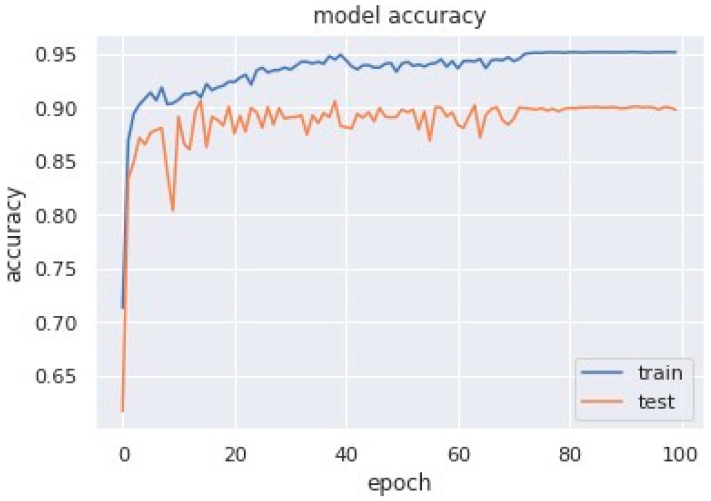
Model accuracy corresponding to epochs (using MobileNet V1).

**Figure 8:**
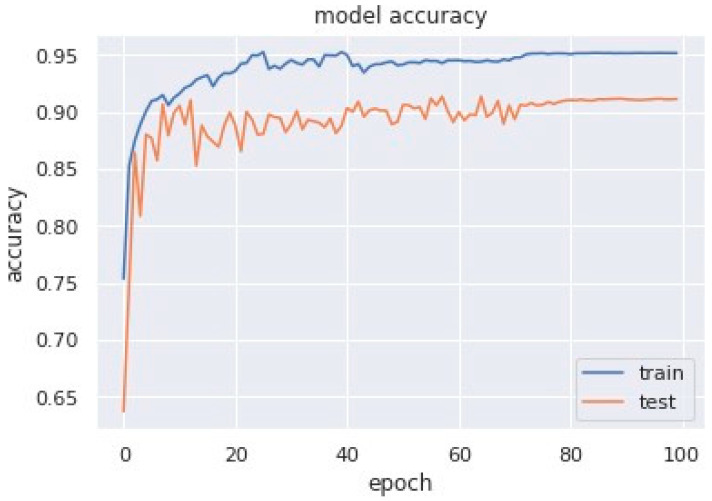
Model accuracy corresponding to epochs (using MobileNet V1 + LSTM).

**Figure 9:**
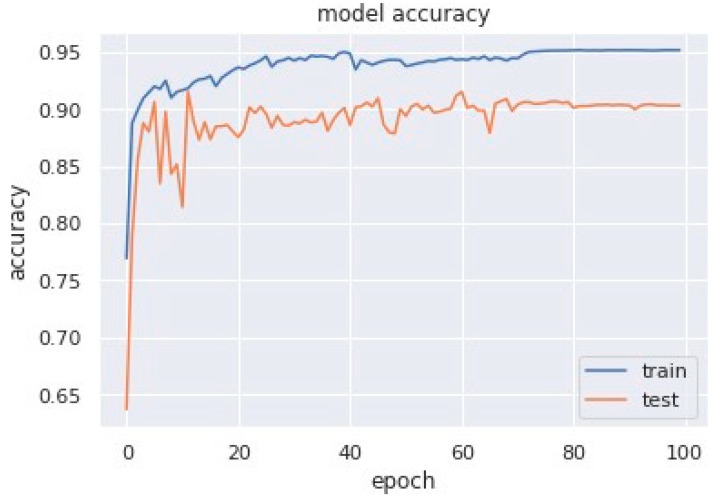
Model accuracy corresponding to epochs (using MobileNet V1 + GRU).

Accuracy graphs are a representation of the model’s performance, based on how much data and experience it is made to work with. The training and testing accuracy are both plotted on the graph to determine the occurrence and severity of overfitting the data; that is, the learning happens to such an extent that it is clearly negatively impacting the accuracy of the results. The gap between the training and testing lines of best fit can be used to check the severity of overfitting. The greater the gap, the fewer epochs should be used to train the model. Figure 6 shows the AUC curve of MobileNet V1 + GRU. Figure 7 shows the model accuracy corresponding to epochs using MobileNet V1 whereas Figure 8 and Figure 9 show the same for MobileNet V1 + LSTM and MobileNet V1 + GRU, respectively. Accuracy graphs of the models proposed in the paper show significant improvements to the existing related works in accordance with better accuracy and low separation between training and testing accuracies. 

**Figure 10:**
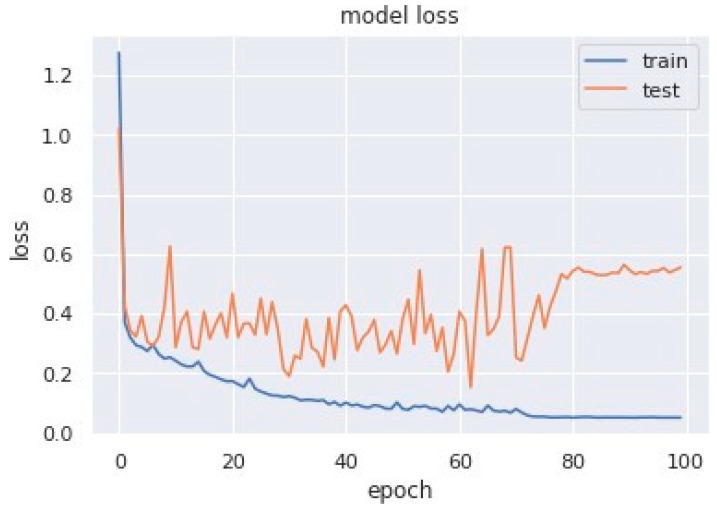
Variation of loss corresponding to epochs (using MobileNet V1).

**Figure 11:**
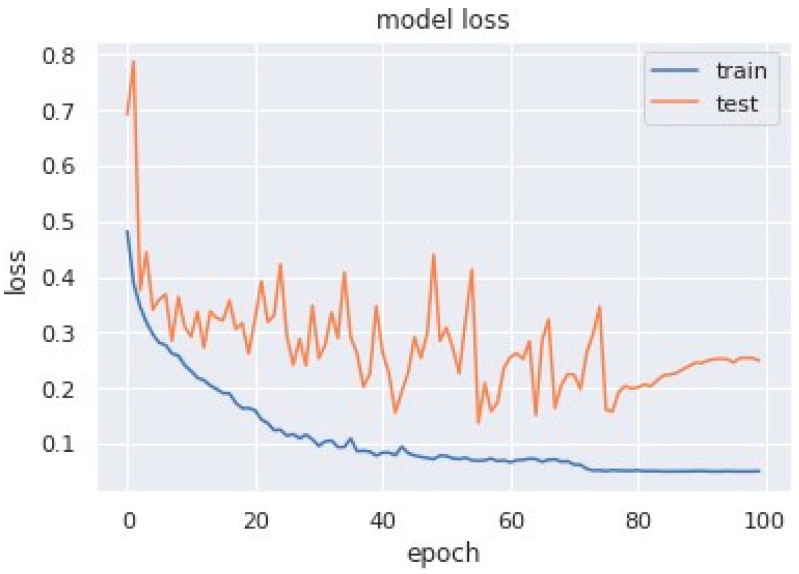
Variation of loss corresponding to epochs (using MobileNet V1 + LSTM).

**Figure 12:**
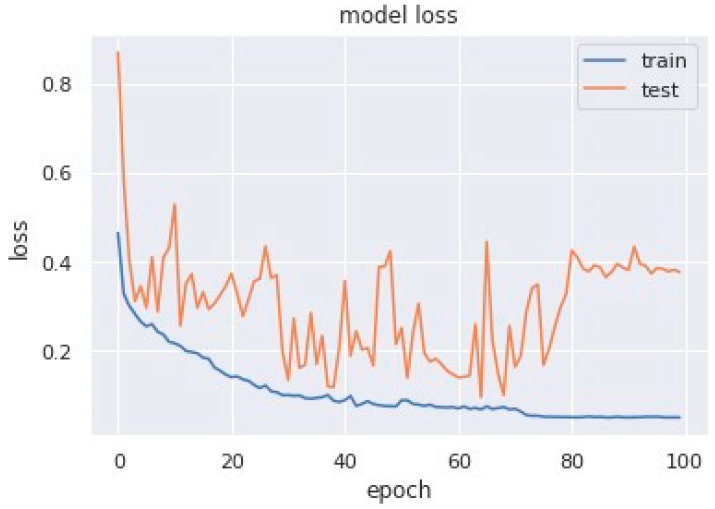
Variation of loss corresponding to epochs (using MobileNet V1 + GRU).

Loss curves are a visual representation of the direction in which the learning of a CNN model takes place, corresponding to the experience and amount of training data it is given. It has an exponentially improving learning rate. Figure 10 shows a variation of loss corresponding to epochs using MobileNet V1. Figure 11 and Figure 12 show a variation of loss corresponding to epochs using MobileNet V1 + LSTM and MobileNet V1 + GRU, respectively. The loss curves of the proposed methods can be seen to become stagnant, parallel and slightly away from each other as the model training comes to an end.

### 4.4. Discussion

Unlike standard image recognition problems, the time series data used in this study had one-dimensional data, which are significantly different from two-dimensional image recognition problems. Compared with the millions of training samples in the field of image classification, the data samples used in this study were smaller, which increases the risk of overfitting. Moreover, Sleep Apnea detection is a binary classification problem that differs from image recognition. The feature maps, convolution layer strides, and fully connected layer nodes in the standard MobileNet V1 may not be suitable for this scene. Therefore, MobileNet V1 is adjusted as follows:

1. A one-dimensional convolution operation is used instead of a two-dimensional convolution operation to feature extraction.
2. A dropout layer between the convolution layer and the fully connected layer is added to avoid over-fitting.
3. Only one fully connected layer is retained so as to reduce network complexity.
4. The size of the convolution layer strides and the number of fully connected layer nodes are modified.

Compared to the standard MobileNet V1, all convolution layer strides of our modified MobileNet V1 were changed to two, and the number of feature maps was increased layer by layer. In particular, a dropout layer with a drop rate of 0.5 was added between the convolution layer and the fully connected layer. In addition, the number of output layer nodes was reduced from 1000 to two for our binary classification problem. Table 9 contains a comparative study of our proposed method and multiple other methods on the Physionet Apnea-ECG database, all of which use Lead-II ECG signals. These studies use a training and testing set of 35 patients each in alignment with our work. From the above comparison of previous studies, we know that [8] has the highest accuracy, specificity and sensitivity of 89.4%, 89.1% and 89.8%, respectively. Whereas the proposed algorithm using MobileNet along with GRU exhibits values higher than those in the previous studies, i.e., accuracy (90.29%), specificity (90.72%), sensitivity (90.01%).

## 5. Wearable Device Implementation (Sleepify)

**Figure 13:**
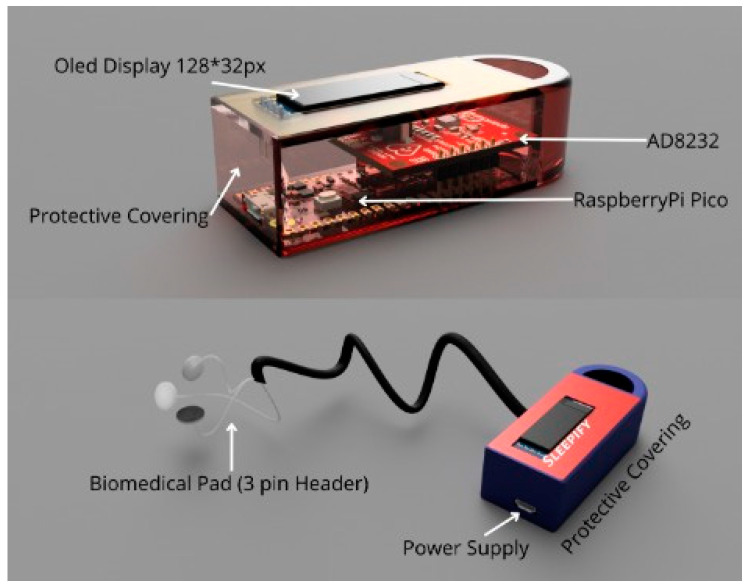
Proposed design for wearable device.

After the proposed models were able to classify the ECG signals into apneic and normal patterns at a decent and acceptable accuracy, the authors went on ahead to resolve the troublesome and tiresome approach of PSG by designing a compact, accurate, and lightweight wearable device named Sleepify. It could be worn by the patients with ease during their sleep at their own homes to record their ECG signals. The device consists of two parts: the first is ECG sensors which will record the ECG signals from the patient, and the other part is raspberry pi pico, which will then diagnose whether the patient is suffered from sleep apnea or not. The device will warn them of any episodes that occurred during the night. The ECG recordings can also be concatenated (upon the patient’s consent) into a new database comprising the annotated ECG signals. The data can be useful to the patient’s diagnosis process and contribute to a new set of databases. For the purpose of recording ECG signals, the signaled data received from the device must be accurate and error-free to one hundred percent. Due to this very reason, the authors have employed security methods in the device’s working; the data can be directly fetched/uploaded from/to the hospital cloud database in encrypted form. Data can also be directly measured from the ECG sensors and then transferred to raspberry pico to classify. Figure 13 shows the design and various components proposed by authors for a secure and compact wearable device. Various tasks such as classification and encryption of the ECG signals for Apnea detection and security applications, respectively, are performed in the microcontroller named, Raspberry Pi Pico.

## 6. Conclusions and Future Scope

The study gives advancement in the area of detection of Obstructive Sleep Apnea using deep learning approaches regarding accuracy, sensitivity and specificity. This study proposes an accurate, cost-effective, and non-invasive methodology for identifying obstructive sleep apnea in potential patients using single-lead ECG signal to train MobileNet V1+ LSTM and MobileNet V1+ GRU, which are CNN-based models with the integration of the RNN model. The proposed model beats the state-of-the-art performance of the existing related studies. The solution requires significantly less computational power and thus can run on portable devices and return results much faster. The proposed model (MobileNet V1 + GRU) achieved an overall accuracy of 90.29% using the sample dataset: a remarkably higher result than other methods and algorithms using the same database and ECG input. Thus, the aforementioned model architecture and its high efficiency make this study and its implementation viable in, for example, portable wearable devices to detect and respond to OSA events in patients, and with the added efficiency, an increase in the speed of diagnosis and detection, given a certain amount of processing power, is naturally observed. In this proposed article, the authors have developed an architecture for deploying a wearable device that can gather data directly from the hospital’s cloud database and utilize that data directly. Further study will involve integrating the device with the hybrid deep learning model and optimizing accuracy. The authors will develop a device that determines the patient’s obstructive sleep apnea diagnosis in real-time.

## Figures and Tables

**Table 3:** Hyperparameter Tuning.

S. No	Hyperparameter	Value
1	Batch Size	32
2	Learning Rate	0.001
3	Epochs	100
4	Loss Function	Categorical Cross Entropy
5	Activation Function	ReLu
6	Optimizer	Adam

**Table 4:** Evaluation analysis for each segment OSA Detection with MobileNet V1.

Spec.	Sens./Rec.	Prec.	F1	Acc.
89	90	89	91	89.5

**Table 5:** Evaluation analysis for each segment OSA Detection with MobileNet V1 + LSTM.

Spec.	Sens./Rec.	Prec.	F1	Acc.
90.3	89.82	94.5	92.1	90

**Table 6:** Evaluation analysis for each segment OSA Detection with MobileNet V1 + GRU.

Spec.	Sens./Rec.	Prec.	F1	Acc.
90.72	90.01	94.71	92.3	90.29

**Table 7:** Comparative analysis for each segment OSA Detection with various models.

Diagnosis	Model 1			Model 2			Model 3		
	Prec.	Rec.	F1-Meas.	Prec.	Rec.	F1-Meas.	Prec.	Rec.	F1-Meas.
Apnea	89	94	91	85	95	92	90.06	94.71	92.33
Normal	90	81	85	90	81	86	90.72	83.18	86.79

**Table 8:** The outcomes of the enhanced Model 2 and Model 3 for per segment classification.

Approach	Sensitivity	Specificity	Accuracy
Model 2	89.82	90.34	90.00
Model 3	90.01	90.72	90.29

**Table 9:** A comparative study between the proposed method and multiple additional methods on the Physionet Apnea-ECG database, using Lead-II ECG signals.

Year	Method	Accuracy	Specificity	Recall/ Sensitivity
2017 [21]	HMM	82.33	84.7	85.8
2018 [9]	DNN and HMM using single lead	85	82.1	88.9
	ECG Signal			
2018 [18]	SVM	88.2	85.7	87.2
2019 [19]	AlexNet model, CNN	86.22	88.4	89
2019 [22]	Time Window artificial neural	87.3	88.7	85.1
	network (TW-MLP)			
2020 [17]	Unsupervised feature learning, single	85.1	86.2	81.4
	lead ECG, HMM			
2021 [8]	Multiscale DNN	89.4	89.1	89.8
Proposed	MobileNet V1	89.5	89	90
Model				
Proposed	MobileNet V1 + LSTM	90.00	90.34	89.82
Model				
Proposed	MobileNet V1 + GRU	90.29	90.72	90.01

## Data Availability

Data available in a publicly accessible repository. The data presented in this study are openly available in [Apnea-ECG Database, Physionet] at [https://doi.org/10.13026/C23W2R], reference number [13].
